# Supplementary material for: Uterine glands coordinate on-time embryo implantation and impact endometrial decidualization for pregnancy success
Source: Nat Commun. 2018 Jun 22;9:2435. doi: 10.1038/s41467-018-04848-8 (PMC6015089; doi:10.1038/s41467-018-04848-8)
Supplement: Supplementary file 1 — Supplementary Information [file 41467_2018_4848_MOESM1_ESM.pdf]

Supplementary Information

Kelleher et al. Uterine glands coordinate on-time embryo implantation and stromal cell decidualization for pregnancy success

Contents:

Supplementary Tables 1 and 2
Supplementary Figures 1-4

Supplementary Table 1. List of Predicted Ligand and Receptor Pairs on GD 4 and their Expression Patterns

	Average FPKM					
	Ligand			Receptor		
**Ligand-Receptor**	**Control**	***Ltf$^{iCre/+}$Foxa2$^{f/f}$***	***Pgr$^{Cre/+}$Foxa2$^{f/f}$***	**Control**	***Ltf$^{iCre/+}$Foxa2$^{f/f}$***	***Pgr$^{Cre/+}$Foxa2$^{f/f}$***
Fn1_Cd44	161.28	127.03	84.93	17.10	16.47	13.21
Fn1_Itga4	161.28	127.03	84.93	2.13	2.34	1.61
Fn1_Itga5	161.28	127.03	84.93	50.79	42.56	32.43
Fn1_Itga6	161.28	127.03	84.93	23.93	19.00	18.69
Fn1_Itga8	161.28	127.03	84.93	3.04	3.54	2.35
Fn1_Itgb8	161.28	127.03	84.93	7.30	9.17	4.54
Fn1_Nt5e	161.28	127.03	84.93	6.29	5.89	2.83
Fn1_Tmprss6	161.28	127.03	84.93	0.35	0.24	0.35
Fn1_Tnfrsf11b	161.28	127.03	84.93	4.54	2.97	5.28
Hp_Asgr1	1.28	3.54	0.28	0.01	0.02	0.05
Hp_Itgam	1.28	3.54	0.28	2.11	1.74	0.99
Hp_Itgb2	1.28	3.54	0.28	7.65	10.01	3.21
Lama2_Itga1	13.98	11.98	7.22	11.64	11.18	8.92
Lama2_Itga6	13.98	11.98	7.22	23.93	19.00	18.69
Lama2_Itga7	13.98	11.98	7.22	10.56	10.37	10.38
Lama2_Itgb4	13.98	11.98	7.22	7.78	10.05	9.03
Sema3C_Nrp1	29.73	29.87	17.61	7.70	5.83	6.30
Ntn1_Neo1	16.18	8.40	8.48	20.31	21.03	14.95
Rgmb_Neo1	20.77	20.84	15.36	20.31	21.03	14.95
Wnt5a_Mcam	28.46	29.63	28.91	77.57	67.14	89.80

*The gene symbols of ligand-receptor pairs are shown in first the column. The mean read counts of ligand and corresponding receptor are shown in the subsequent columns.

Supplementary Table 2. Antibody information

Antibody	Type	Manufacturer	Catalog no.	Dilution	Application[2]
CDH1	Rabbit IgG	Thermo Fisher Scientific	ECCD-2	1:400	IF
CK8	Rat IgG	DSHB[1]	-	1:50	IF
CLDN1	Rabbit IgG	Thermo Fisher Scientific	MH25	1:600	IF
FOXA2	Rabbit IgG	Abcam	ab108422	1:1000	IF
Ki67	Rabbit IgG	Abcam	ab15580	1:1000	IF
p-Tyr705 STAT3	Rabbit IgG	Bioworld Technology	BS4181	1:300	IF
PTGS2	Rabbit IgG	Cayman Chemicals	160106	1:250	IF/IHC

[1]DSHB, University of Iowa Developmental Studies Hybridoma Bank
[2]IF, immunofluorescence; IHC, immunohistochemistry;

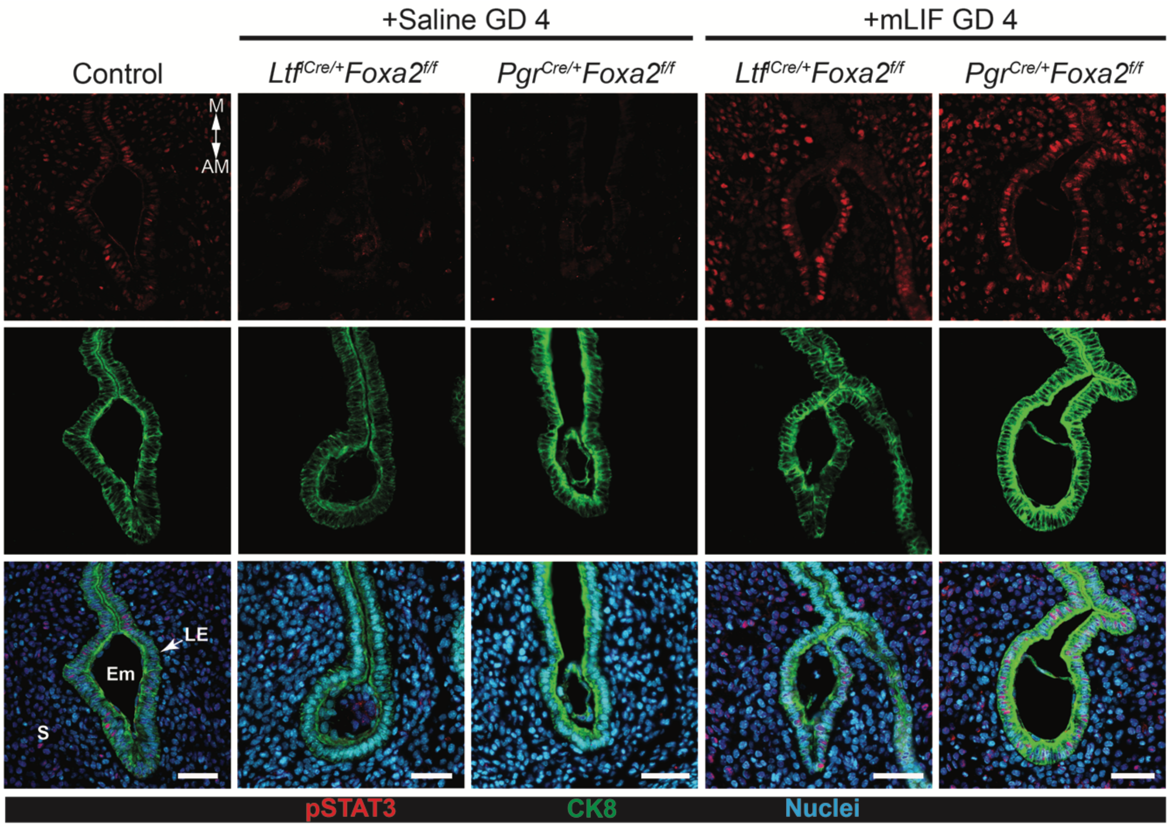

Supplementary Figure 1 | Effects of LIF on STAT3 localization in implantation sites on GD 4. Treatment with LIF elicited an increased in pSTAT3 levels in the LE and stroma in both types of FOXA2-deficient mice. Scale bar: 50 μm. AM antimesometrial, M mesometrial, Em embryo, LE luminal epithelium, S stroma. All images are representative of three independent experiments.

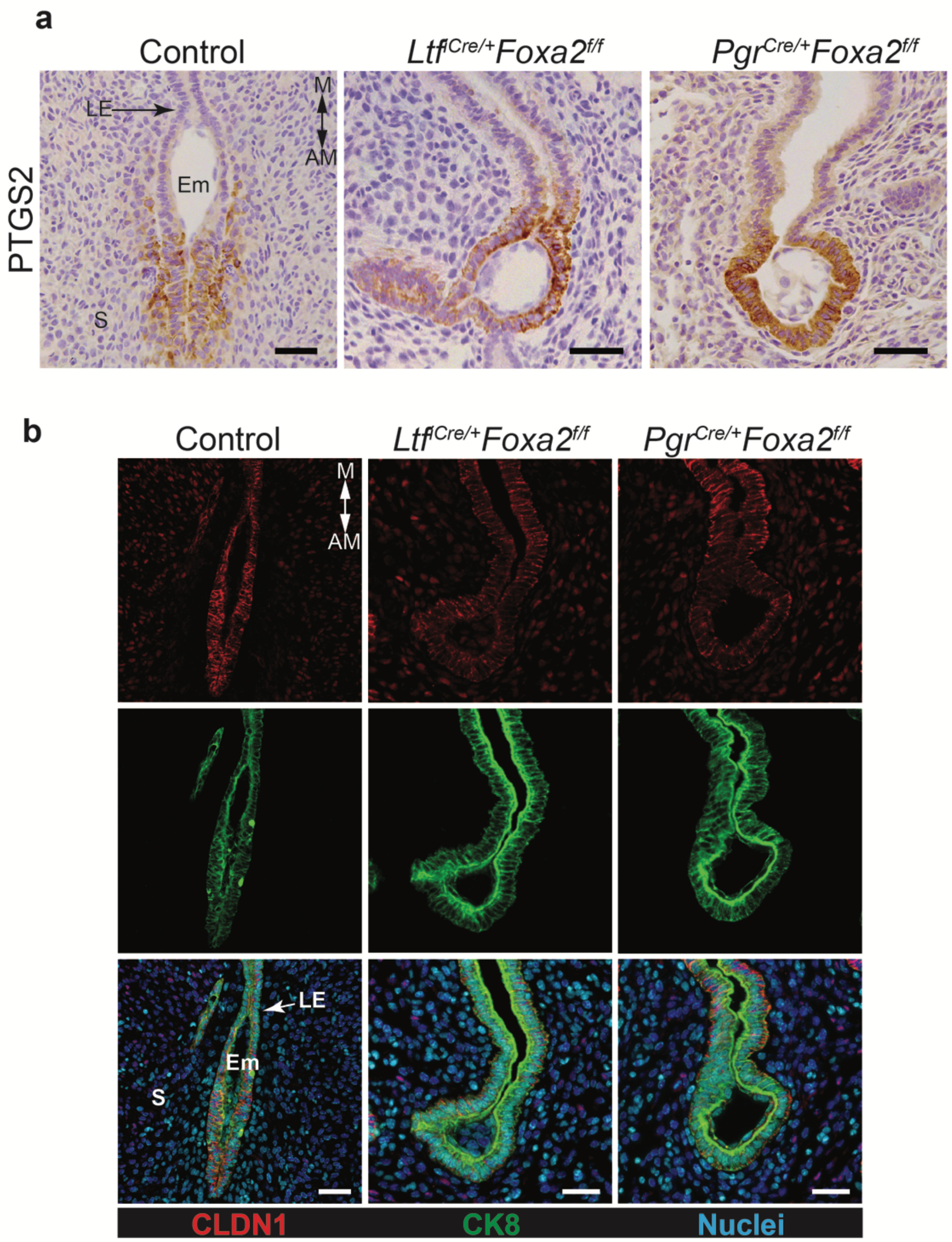

Supplementary Figure 2 | Assessment of epithelial remodeling and stromal cell decidualization on GD 5 in FOXA2-deficient mice without LIF repletion. a, Immunolocalization of PTGS2 at 0900 h on GD 5 in implantation sites from control and FOXA2-deficient mice. Note that PTGS2 is present in the decidualizing stroma of control mice but is only in the LE adjacent to the blastocyst in FOXA2-deficient mice. Scale bar: 100 μm. **b,** Immunofluorescence analysis of CLDN1 in implantation sites. Scale bar: 50 μm. AM antimesometrial, M mesometrial, Em embryo, LE luminal epithelium, S stroma. All images are representative of three independent experiments.

a

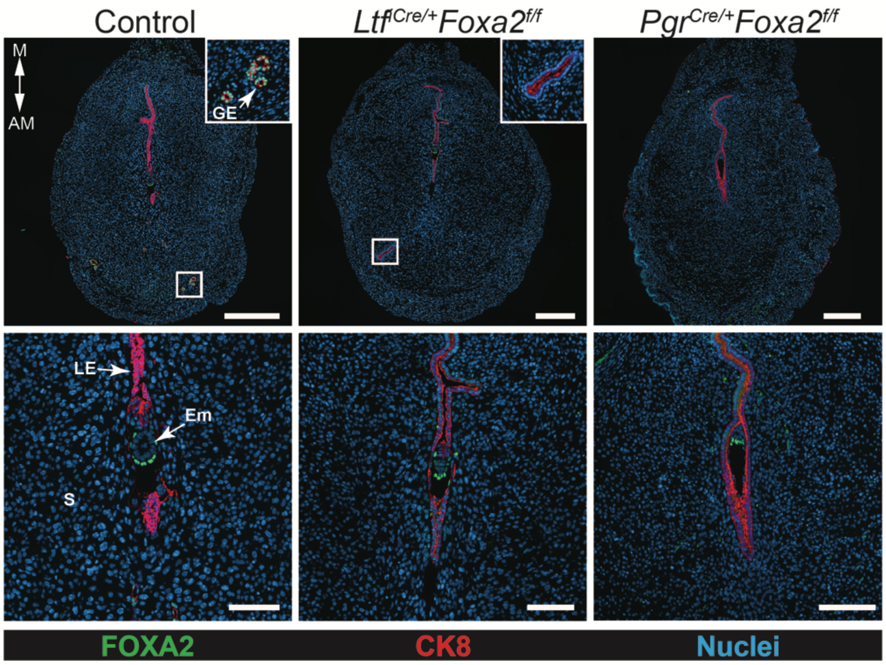

b

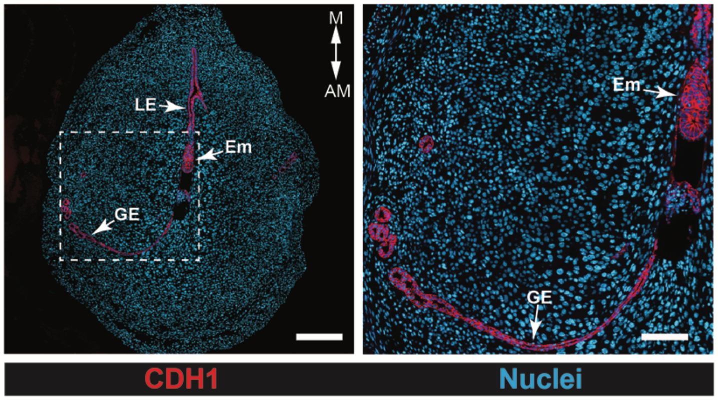

Supplementary Figure 3 | Uterine glands are present in the implantation site on GD 5. a, Immunoreactive distribution of FOXA2 in implantation sites on GD 5 at 2000 h. FOXA2-deficient mice received i.p. injections of LIF on GD 4. Note nuclear FOXA2 in the glands and embryonic endoderm in control mice, but no FOXA2 in the CK8-positive glands in $Ltf^{iCre/+}Foxa2^{f/f}$ mice. Row 1- Scale bar: 250 µm; Row 2- Scale bar: 100 µm. Em embryo, GE glandular epithelium, LE luminal epithelium. **b,** Immunofluoresence analysis of CDH1 in control mice demonstrates that glands directly connect into the implantation chamber. Left panel- Scale bar: 250 µm; Right panel- Scale bar: 100 µm. AM antimesometrial, M mesometrial, Em embryo, GE glandular epithelium, LE luminal epithelium. All images are representative of three independent experiments.

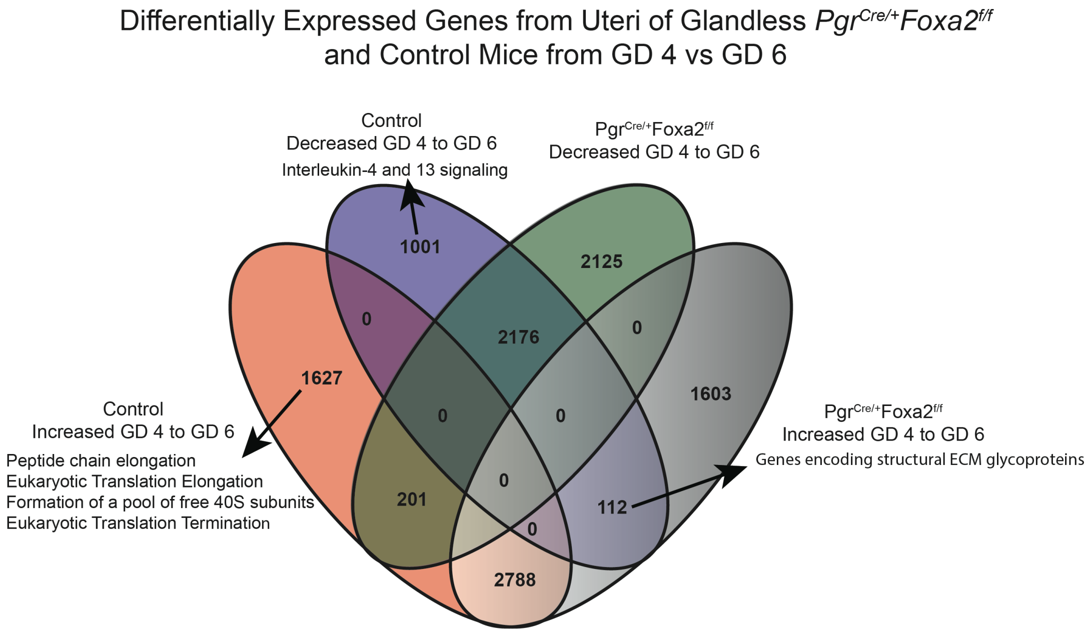

Supplementary Figure 4 | Venn diagrams of differentially expressed genes in uteri of LIF-repleted glandless *Pgr$^{Cre/+}$Foxa2$^{f/f}$* and control mice from GD 4 and GD 6. Selected pathways enriched in the gene sets were determined by functional analysis.
