# Supplementary material for: Uterine glands coordinate on-time embryo implantation and impact endometrial decidualization for pregnancy success
Source: Nat Commun. 2018 Jun 22;9:2435. doi: 10.1038/s41467-018-04848-8 (PMC6015089; doi:10.1038/s41467-018-04848-8)
Supplement: Supplementary file 3 — Description of Additional Supplementary Files [file 41467_2018_4848_MOESM3_ESM.pdf]

**Description of Additional Supplementary Files**

File Name: **Supplementary Data 1**
Description: Differentially Expressed Genes in GD 4 Uteri from Control and *Pgr*$^{Cre/+}$*Foxa2*$^{f/f}$ Mice

File Name: **Supplementary Data 2**
Description: Differentially Expressed Genes in GD 4 uteri from Control and *Ltf*$^{iCre/+}$*Foxa2*$^{f/f}$ Mice

File Name: **Supplementary Data 3**
Description: Gland-enriched Genes Uniquely Differentially Expressed in uteri of *Pgr*$^{Cre/+}$*Foxa2*$^{f/f}$ Mice on GD 4

File Name: **Supplementary Data 4**
Description: TOPPFUN Analysis of Gland-enriched Genes Uniquely Differentially Expressed in uteri of *Pgr*$^{Cre/+}$*Foxa2*$^{f/f}$ Mice on GD 4

File Name: **Supplementary Data 5**
Description: Differentially Expressed Genes in GD 6 Implantation Sites from Control and LIF-Replaced *Pgr*$^{Cre/+}$*Foxa2*$^{f/f}$ Mice

File Name: **Supplementary Data 6**
Description: TOPPFUN Analysis of Differentially Expressed Genes from Implantation Sites of LIF-replaced *Pgr*$^{Cre/+}$*Foxa2*$^{f/f}$ and Control Mice on GD 6

File Name: **Supplementary Data 7**
Description: Differentially Expressed Genes from GD 4 and GD 6 in Control Mice

File Name: **Supplementary Data 8**
Description: Differentially Expressed Genes from GD 4 and GD 6 in *Pgr*$^{Cre/+}$*Foxa2*$^{f/f}$ Mice

File Name: **Supplementary Data 9**
Description: TOPPFUN Analysis of Genes Increased in Uteri from GD 4 to GD 6 in Control Mice

File Name: **Supplementary Data 10**
Description: TOPPFUN Analysis of Genes Decreased in Uteri from GD 4 to GD 6 in Control Mice

File Name: **Supplementary Data 11**
Description: TOPPFUN Analysis of Genes Increased from GD 4 to GD 6 in $Pgr^{Cre/+}Foxa2^{f/f}$ Mice

File Name: **Supplementary Data 12**
Description: TOPPFUN Analysis of Genes Decreased from GD 4 to GD 6 in $Pgr^{Cre/+}Foxa2^{f/f}$ Mice

File Name: **Supplementary Data 13**
Description: Genes Increased in Uteri of LIF-replaced $Pgr^{Cre/+}Foxa2^{f/f}$ Mice, but Declined in the Uteri of Control Mice Between GD 4 and GD 6

File Name: **Supplementary Data 14**
Description: TOPPFUN Analysis of Genes Increased in Uteri of LIF-replaced $Pgr^{Cre/+}Foxa2^{f/f}$ Mice, but Declined in the Uteri of Control Mice Between GD 4 and GD 6
